# Generalized reporter score-based enrichment analysis for omics data

**DOI:** 10.1093/bib/bbae116

**Published:** 2024-03-27

**Authors:** Chen Peng, Qiong Chen, Shangjin Tan, Xiaotao Shen, Chao Jiang

**Affiliations:** MOE Key Laboratory of Biosystems Homeostasis & Protection, and Zhejiang Provincial Key Laboratory of Cancer Molecular Cell Biology, Life Sciences Institute, Zhejiang University, Hangzhou, Zhejiang 310030, China; State Key Laboratory for Diagnosis and Treatment of Infectious Diseases, First Affiliated Hospital, Zhejiang University School of Medicine, Hangzhou, Zhejiang 310009, China; MOE Key Laboratory of Biosystems Homeostasis & Protection, and Zhejiang Provincial Key Laboratory of Cancer Molecular Cell Biology, Life Sciences Institute, Zhejiang University, Hangzhou, Zhejiang 310030, China; State Key Laboratory for Diagnosis and Treatment of Infectious Diseases, First Affiliated Hospital, Zhejiang University School of Medicine, Hangzhou, Zhejiang 310009, China; BGI Research, Wuhan, Hubei 430074, China; BGI Research, Shenzhen, Guangdong 518083, China; Department of Genetics, Stanford University School of Medicine, Stanford, CA, USA; MOE Key Laboratory of Biosystems Homeostasis & Protection, and Zhejiang Provincial Key Laboratory of Cancer Molecular Cell Biology, Life Sciences Institute, Zhejiang University, Hangzhou, Zhejiang 310030, China; State Key Laboratory for Diagnosis and Treatment of Infectious Diseases, First Affiliated Hospital, Zhejiang University School of Medicine, Hangzhou, Zhejiang 310009, China; Center for Life Sciences, Shaoxing Institute, Zhejiang University, Shaoxing, Zhejiang 321000, China

**Keywords:** reporter score, enrichment analysis, multi-group, omics data, customized database

## Abstract

Enrichment analysis contextualizes biological features in pathways to facilitate a systematic understanding of high-dimensional data and is widely used in biomedical research. The emerging reporter score-based analysis (RSA) method shows more promising sensitivity, as it relies on *P*-values instead of raw values of features. However, RSA cannot be directly applied to multi-group and longitudinal experimental designs and is often misused due to the lack of a proper tool. Here, we propose the Generalized Reporter Score-based Analysis (GRSA) method for multi-group and longitudinal omics data. A comparison with other popular enrichment analysis methods demonstrated that GRSA had increased sensitivity across multiple benchmark datasets. We applied GRSA to microbiome, transcriptome and metabolome data and discovered new biological insights in omics studies. Finally, we demonstrated the application of GRSA beyond functional enrichment using a taxonomy database. We implemented GRSA in an R package, ReporterScore, integrating with a powerful visualization module and updatable pathway databases, which is available on the Comprehensive R Archive Network (https://cran.r-project.org/web/packages/ReporterScore). We believe that the ReporterScore package will be a valuable asset for broad biomedical research fields.

## Graphical Abstract

**Graphical Abstract:**
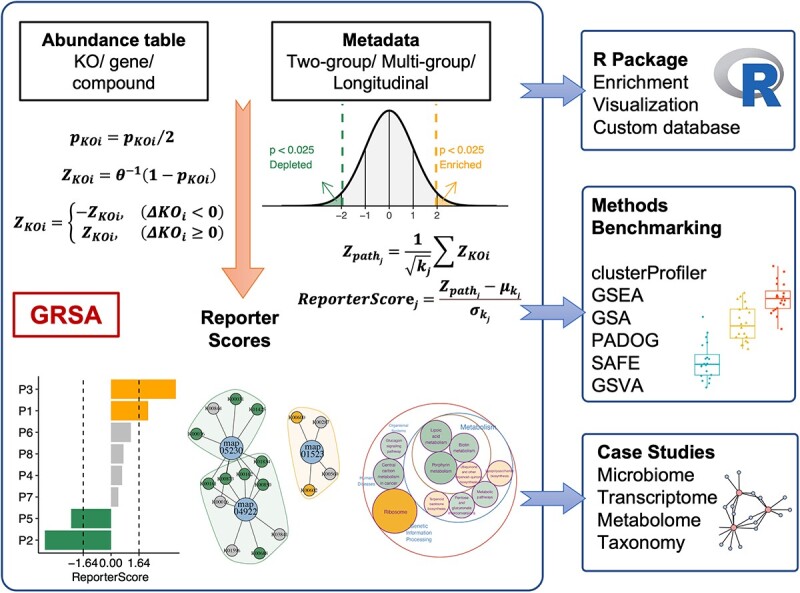

## INTRODUCTION

Functional enrichment analysis is a popular bioinformatic method that helps understand the biological significance of large omics datasets, such as transcriptomic, metagenomic, and metabolic data. We gain insights into the underlying biological processes and functions by identifying enriched functional categories, such as gene ontology terms or biological pathways, and formulate hypotheses for downstream experimental investigations [1].

Methods for functional enrichment analysis can be roughly divided into three categories based on underlying statistical methods: (i) overrepresentation analysis (ORA), (ii) functional class scoring (FCS) and (iii) pathway topology-based (PT) [2]. Common enrichment analysis methods in omics research are shown in Table 1. In addition, Goeman and Bühlmann classified enrichment analysis methods based on the underlying null hypothesis as ‘competitive’ or ‘self-contained’. In ‘competitive’ methods, a gene set is compared against the background of all genes not in the set to assess if the level of statistical differences exceeds the background level, whereas a ‘self-contained’ method analyzes each gene set in isolation [11].

**Table 1:** Some commonly used enrichment analysis methods

Category	Method	Tools	Notes
ORA	Hypergeometric test/Fisher’s exact test	DAVID (website) [3], clusterProfiler (R package) [4]	The most common methods used in enrichment analysis. Selecting a list of genes is required
FCS	Gene set enrichment analysis (GSEA)	GSEA (website) [5]	GSEA creatively uses gene ranking, rather than selecting a list of genes, to identify statistically significant and concordant differences across gene sets
FCS	Generalized reporter score-based analysis (GRSA/RSA)	ReporterScore (R package developed in this study)	Find significant metabolites (first report), pathways and taxonomy based on the *P*-values for multi-omics data
FCS	Significance Analysis of Function and Expression (SAFE)	SAFE (R package) [6]	SAFE assesses the significance of gene categories by calculating both local and global statistics from gene expression data
FCS	Gene Set Analysis (GSA)	GSA (R Package) [7]	GSA was proposed as an improvement of GSEA, using the ‘maxmean’ statistic instead of the weighted sign KS statistic
FCS	Pathway Analysis with Down-weighting of Overlapping Genes (PADOG)	PADOG (R package) [8]	PADOG assumes that genes associated with fewer pathways have more significant effects than genes associated with more pathways
FCS	Gene Set Variation Analysis (GSVA)	GSVA (R package) [9]	A non-parametric, unsupervised method that transforms gene expression data into gene set scores for downstream differential pathway activity analysis
PT	Topology-based pathway enrichment analysis (TPEA)	TPEA (R package) [10]	Integrate topological properties and global upstream/downstream positions of genes in pathways

The algorithm of reporter score-based analysis (RSA) was originally developed by Patil and Nielsen in 2005 to identify metabolites associated with the metabolic network’s regulatory hotspots [12]. The RSA has recently gained popularity due to its extended application in functional enrichment analysis in microbiome research [13]. RSA is a competitive FCS method based on parsing the *P*-values of selected statistical analyses without a priori cut-off (threshold-free). The rationale is that the *P*-value can be considered a standardized statistic reflecting the differences between different genes or features, regardless of the mean expression values. The pathways with significantly lower *P*-values than the background *P*-value distribution are enriched [12].

However, RSA is often misused due to a lack of specific tools and a systematic understanding of the algorithm [14]. In addition, the sign (plus or minus) of the reporter score of each pathway in classic RSA does not represent the increasing or decreasing trend of the pathway expression; rather, reporter scores (including negative value) less than a specified threshold indicate that the corresponding pathway is not significantly enriched. This often leads to misinterpretations of the results.

Inspired by the classic RSA, we developed the improved Generalized Reporter Score-based Analysis (GRSA) method, implemented in the R package ReporterScore, along with comprehensive visualization methods and pathway databases. GRSA is a threshold-free method that works well with all types of biomedical features, such as genes, chemical compounds and microbial species. GRSA works in the mixed (classic RSA) and directed modes (enhanced RSA). The directed mode uses signs of the reporter score to distinguish up-regulated or down-regulated pathways, which is more intuitive. Importantly, the GRSA supports multi-group and longitudinal experimental designs because of the included multi-group-compatible statistical methods (for a full list of supported methods, please see Supplementary Table S1). The ReporterScore package also supports custom hierarchical and relational databases (e.g. tables containing the correspondence between pathways and genes), providing extra flexibility for advanced users. In this study, we described the comprehensive utility of GRSA. We benchmarked GRSA against other popular enrichment methods across multiple datasets and demonstrated the applications of GRSA on diverse omics datasets.

## MATERIALS AND METHODS

### Algorithm

The algorithm of GRSA is described as follows, using metagenomic data as an example.

#### (1) Calculating the *P*-values

**Figure 1:**
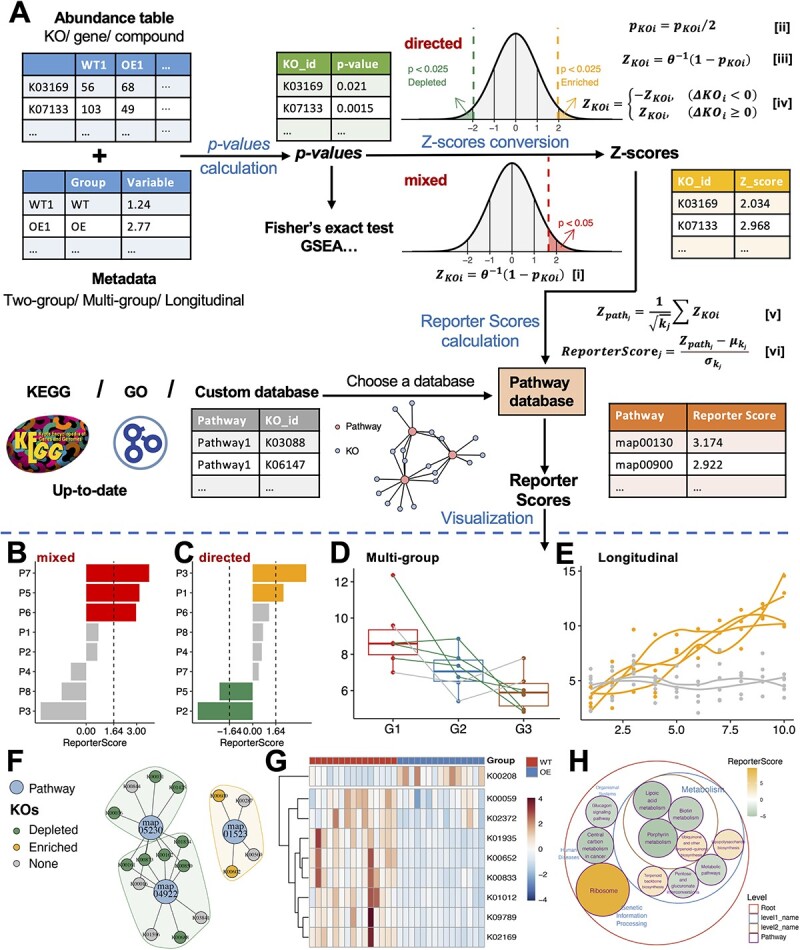
The overall workflow of GRSA in the ReporterScore package. (**A**) GRSA workflow consists of four parts: Calculate the *P*-values for KOs between two or multiple groups by various statistical methods; convert the *P*-values of KOs to *Z*-scores by inverse normal distribution and assignment of a plus or minus sign to each *Z*-score in the directed mode; mapping KOs to annotated pathways and calculating the reporter score for each pathway; visualize the results of GRSA. $K{O}_i$ represents a certain KO; ${p}_{K{O}_i}$ is the *P*-value of $K{O}_i$; ${Z}_{K{O}_i}$ is the *Z*-score transformed from ${p}_{K{O}_i}$; $\varDelta K{O}_i$ is the abundance difference of between groups. A total of ${k}_j$ KOs were annotated to the corresponding pathway. ${\mu}_{k_j}$ and ${\sigma}_{k_j}$ are the mean and the standard deviation of the background *Z*-score distribution ${Z}_{path\_ null}$, respectively. The ReporterScore package provides various visualization methods for the GRSA result. (**B**) The bar chart shows reporter scores of pathways in the mixed mode. The red color indicates significantly enriched pathways, with reporter scores greater than 1.64, corresponding to a *P*-value of 0.05. (**C**) The bar chart shows reporter scores of pathways in the directed mode. The orange and green colors indicate up-regulated and down-regulated pathways with absolute reporter scores >1.64. (**D** and **E**) The line charts show the pattern of a selected pathway in the directed mode with a multi-group (**D**) and a longitudinal design (**E**), each line represents the trend of the average abundance of one KO. Line colors indicate whether the KO is significantly enriched (orange), depleted (green) or neither (gray). (**F**) The network plot shows the KOs present in selected pathways; some KOs can be shared by several pathways. Big dots represent pathways, and small dots represent KOs. The colors of small dots represent the trend of KOs. The colors of the shades encircling pathways denote whether the pathway is overall up-regulated (orange) or down-regulated (green). (**G**) The heatmap displays the abundance of each KO in a pathway for different samples (columns). (**H**) The circular packing plot shows the hierarchical relationship of selected pathways; the size of the circle indicates the absolute value of the reporter score, and the color of the circle indicates that the pathway is overall up-regulated (orange) and down-regulated (green).

A statistical method (the full list of supported statistical methods is in Supplementary Table S1) was used to obtain the *P*-values of the features (i.e. ${p}_{K{O}_i}$, $K{O}_i$ represents a certain KO; Figure 1A). We used KO to represent different features in the formulas for simplicity.

#### (2) Converting the *P*-values into *Z*-scores

For the classic mixed RSA, we used an inverse normal cumulative distribution function (${\theta}^{-1}$) to convert the *P*-values of KOs into *Z*-scores. Thus, assuming uniformly distributed *P*-values under the random data assumption ranges, the resulting *Z*-scores will follow a standard normal distribution (Figure 1A), and the formula is


(1) \begin{equation*} {Z}_{K{O}_i}={\theta}^{-1}\left(1-{p}_{K{O}_i}\right) \end{equation*}

For the new directed RSA, we first divided the *P*-values by 2, transforming the range of *P*-values from $\left(0,1\right]$ to $\left(0,0.5\right]$ :


(2) \begin{equation*} {p}_{K{O}_i}={p}_{K{O}_i}/2\kern2.00em \end{equation*}

Secondly, we used an inverse normal cumulative distribution function to convert the *P*-values of KOs into *Z*-scores (equation (1)). When the *P*-value is 0.5, the converted *Z*-score equals 0. Since the above *P*-values are no greater than 0.5, all converted *Z*-scores will be >0 (Figure 1A).

We then determined if a KO is up-regulated or down-regulated and calculated the $\varDelta K{O}_i$.

In a differential abundance analysis (two-group design):


(3) \begin{equation*} \varDelta K{O}_i=\overline{K{O}_{i_{g1}}}-\overline{K{O}_{i_{g2}}} \end{equation*}

$\overline{K{O}_{i_{g1}}}$ is the average abundance of $K{O}_i$ in group 1, and $\overline{K{O}_{i_{g2}}}$ is the average abundance of $K{O}_i$ in group 2.

In a correlation analysis (two-group, multi-group and longitudinal design):


(4) \begin{equation*} \varDelta K{O}_i={\rho}_{K{O}_i} \end{equation*}

${\rho}_{K{O}_i}$ is the correlation coefficient between $K{O}_i$ and the numeric variable.

Finally, assign a plus or minus sign to each *Z*-score:


(5) \begin{equation*} {Z}_{K{O}_i}=\left\{\begin{array}{@{}cc}-{Z}_{K{O}_i},& \left(\varDelta K{O}_i<0\right)\\{}+{Z}_{K{O}_i},& \left(\varDelta K{O}_i\ge 0\right)\end{array}\right. \end{equation*}

Therefore, a $K{O}_i$ with a ${Z}_{K{O}_i}$ >0 is up-regulated and a $K{O}_i$ with a ${Z}_{K{O}_i}$ <0 is down-regulated.

#### (3) Scoring the pathway

We next used the *Z*-scores of KOs to score the pathway. First, choose a pathway database as the reference. It is of particular interest to note any hierarchy relational table (e.g. KEGG, taxonomy database) can be used as a reference as long as the relationship between the upstream and downstream features (e.g. pathways and KOs) can be represented by a bipartite network (Figure 1A). For each pathway in the selected database, calculate the *Z*-score of pathway *j* (${Z}_{pat{h}_j}$) as follows:


(6) \begin{equation*} {Z}_{pat{h}_j}=\frac{1}{\sqrt{k_j}}\sum_{i=1}^{k_j}{Z}_{K{O}_i} \end{equation*}

where ${Z}_{K{O}_i}$ is the *Z*-score of $K{O}_i$ within $pat{h}_j$, and ${k}_j$ denotes the total number of KOs in $pat{h}_j$.

Next, we corrected ${Z}_{pat{h}_j}$ using the randomly sampled ${Z}_{path\_ nul{l}_j}$ from the background distribution of the *Z*-scores of all KOs (${Z}_{K{O}_{all}}=\big\{{Z}_{K{O}_1},{Z}_{K{O}_2},\dots, {Z}_{K{O}_j}\big\}$) to evaluate the significance of enrichment. Specifically, for a given pathway $pat{h}_j$ including ${k}_j$ KOs, we randomly sampled the same number ($j$) of KOs from the background ${Z}_{K{O}_{all}}$ without replacement, and calculated the ${Z}_{path\_ nul{l}_j}$, for *N* times (*N* = 10 000 in this study [15]). We then standardized ${Z}_{pat{h}_j}$ by subtracting the mean (${\mu}_{k_j}$) and dividing by the standard deviation (${\sigma}_{k_j}$) of the ${Z}_{path\_ nul{l}_j}$ distribution. The standardized ${Z}_{pat{h}_j}$ is the $ReporterScor{e}_j$. The *P*-value of $ReporterScor{e}_j$ is also estimated by the above permutation. The formulas for the reporter scores and associated *P*-values are


(7) \begin{equation*} ReporterScor{e}_j=\frac{Z_{pat{h}_j}-{\mu}_{k_j}}{\sigma_{k_j}} \end{equation*}

(8) \begin{equation*} p\_ valu{e}_j=\frac{\sum_{n=1}^NI\left({Z}_{pat{h}_j},{Z}_{pat h\_ nul{l}_{jn}}\right)+1}{N+1}\kern1em \end{equation*}

where ${Z}_{path\_ nul{l}_j}$ have the same $k$ to ${Z}_{pat{h}_j}$, ${\mu}_{k_j}$ is the mean of the randomly generated *N*${Z}_{path\_ nul{l}_j}$ and ${\sigma}_{k_j}$ is the standard deviation of the randomly generated *N*${Z}_{path\_ nul{l}_j}$.

For the classic mixed RSA:


(9) \begin{equation*} I\left(a,b\right)=\left\{\begin{array}{@{}cc}1& \left(a\ge b\right)\\{}0& \left(a<b\right)\end{array}\right. \end{equation*}

For the new directed RSA:


(10) \begin{equation*} I\left(a,b\right)=\left\{\begin{array}{@{}cc}1& \left( ReporterScor{e}_j\ge 0\&a\ge b\right)\\{}1& \left( ReporterScor{e}_j<0\&a\le b\right)\\{}0& \left(\mathrm{Otherwise}\right)\end{array}\right. \end{equation*}

### Statistical analyses

All statistical analyses were done on the R 4.2.2 platform. The developed ReporterScore package (https://cran.r-project.org/web/packages/ReporterScore/) was used for GRSA and visualization. The Venn diagram and Venn network diagram were drawn by the pcutils package (https://cran.r-project.org/web/packages/pcutils/). We collected several datasets for benchmarking and three different type omics datasets as case study [16–18]. Details of these datasets can be found in Supplementary Notes.

To compare the performance of different statistical methods in the two-group experimental design, we defined a Jaccard similarity index:


(11) \begin{equation*} \mathrm{Similarity}=\frac{\left| method(i)\cap method(j)\right|}{\left| method(i)\cup method(j)\right|}\kern2.00em \end{equation*}

where *method*(*i*) (*method*(*j*)) is the number of significant pathways based on benchmark data sets enriched by different methods.

We used fuzzy c-means (FCM) clustering to explore the performance of different statistical test methods in multi-group experimental design (Supplementary Figure S2B,C) and gene expression patterns in transcriptome (Figure 4A). FCM is an unsupervised machine-learning technique that partitions a population into groups or clusters [19]. Three methods (Elbow, Silhouette and Gap statistic) were used to determine the optimal number of clusters. In FCM, the membership score is the probability that a feature belongs to a cluster. Each feature is assigned to a cluster based on its highest membership score.

### Comparison with other enrichment analysis methods

We compared GRSA against six commonly used enrichment analysis methods (Table 1) using 50 benchmark datasets (41 human gene expression datasets related to disease and 9 wild-type/knockout mice gene expression datasets).

We first calculated the *P*-values of features by *t*-test and performed adjustment of the *P*-values using the Benjamini and Hochberg (BH) method to control for false discovery rate. In order to perform the overrepresentation analysis, we selected the genes that have *P*-values <0.05. The top 400 genes with the highest unsigned log-fold changes were considered as differentially expressed genes (DEGs) [20]. Improved fisher.test (CP) was performed by enricher function in the clusterProfiler package with the DEGs list. Gene set enrichment analysis (GSEA) was performed by the GSEA function in the clusterProfiler package, and the *t*-test statistic was used as the metric for ranking [21]. The R packages safe, GSVA, GSA and PADOG were used to run the respective methods. GRSA and RSA were performed with the BH-adjusted *P*-values of features by the ReporterScore package in the directed and mixed mode, respectively. A number of 1000 permutations were used in all methods relying on sample or gene permutations.

Each human gene expression dataset in SupplementaryTable S2 was associated with a disease with the defined mechanism in a KEGG pathway (termed the target pathway). Ideally, a good enrichment analysis method would rank the target pathway at or near the top of the list with a small adjusted *P*-value. Our human gene expression datasets were sourced from a list provided by Nguyen et al. [22]. Based on statistical and biological considerations, we selected datasets in which the number of samples was consistent between the control and treatment groups (<2× difference in sample size is allowed). Furthermore, there should be a measurable overall statistical difference between the gene expressions of the two groups (Adonis *P*-value <0.05). The final 24 benchmark datasets are related to 10 human diseases, and each disease is associated with 2–4 datasets. For further benchmarking analysis, we also included 32 datasets provided by the ‘GSEABenchmarkeR’ package [23].

For wild-type/knockout mice gene expression datasets (Supplementary Table S3), we searched the GEO database for mouse single-gene knockout experiments. We selected datasets where more than three pathways were impacted, as this provides a robust basic for benchmarking various methods. We considered the pathways containing the knockout gene as true positives and the pathways without the knockout gene as true negatives [22]. An adjusted *P*-value threshold of 0.05 was used to determine if a pathway is significantly affected. Based on the definitions of true positives (TP), true negatives (TN), false positives (FP) and false negatives (FN), we can calculate the sensitivity and specificity as follows:


(12) \begin{equation*} \mathrm{Sensitivity}=\frac{TP}{TP+ FN}\kern1em \end{equation*}

(13) \begin{equation*} \mathrm{Specificity}=\frac{TN}{TN+ FP}\kern1em \end{equation*}

## RESULTS

### Workflow overview

The ReporterScore package has built-in KEGG pathway, module, gene, compound and GO databases and also allows customized databases, making it compatible with feature abundance tables from diverse omics data. A complete gene abundance table can be used for transcriptomic, scRNA-seq and related gene-based omics data of a specific species. For metagenomic and metatranscriptomic data, which involve many different species, a KO abundance table can be used, generated using Blast, Diamond or KEGG official mapper software [24] to align the reads or contigs to the KEGG [25] or the EggNOG database [26]. An annotated compound abundance table can be used for metabolomic data, but the standardization of compound IDs (e.g. convert compound IDs to KEGG IDs) is required.

The workflow of GRSA in the ReporterScore package is shown in Figure 1, using metagenomic data as an example. The KO abundance table (rows are KOs and columns are samples) and metadata table (rows are samples and columns are experimental design groups) were used as the input for GRSA. Importantly, the input data should not be prefiltered to retain the background information. First, the *P*-values for all KOs were calculated by a proper statistical method. Then, in the classic mode, the *P*-values were directly converted to *Z*-scores (Figure 1A, equation [i]). In the directed mode, the *P*-values were divided by 2, converted to *Z*-scores, and assigned plus or minus signs, denoting up- and down-regulated KOs (Figure 1A, equations [ii–iv]). Next, the *Z*-score of pathway $j$ (${Z}_{pat{h}_j}$) was calculated by summing the *Z*-scores of KOs within pathway $j$ and dividing by the square root of the number of KOs (${k}_j$) in pathway $j$ (Figure 1A, equation [v]). The ${Z}_{pat{h}_j}$ is further standardized by the background pathway *Z*-score distribution, generated by randomly sampling ${k}_j$ KOs from the total KO pool (Figure 1A, equation [vi]). The standardized pathway *Z*-score is defined as the reporter score of a pathway ($ReporterScor{e}_j$).

We designed the ReporterScore package to be user-friendly. In one step, the function reporter_score calculates the reporter scores for a feature abundance table with associated metadata. The included assorted visualization methods can be used to explore the entire pathways and features within pathways (Figure 1B–H). The demo code is included in the Supplementary Notes.

### Applying GRSA to multi-group and longitudinal omics data

An important feature of GRSA is the newly developed directed mode. The key difference between the directed mode and the mixed mode (classic RSA) is that in the directed mode, the reporter score’s plus or minus sign indicates the pathway’s increasing or decreasing trend (Figure 1C). However, in the mixed mode, the signs of the reporter score do not indicate the trends of the pathways. We performed GRSA on the public ex_KO_profile dataset (a metagenomic dataset) in two modes (Supplementary Figure S1A). For pathways enriched in the directed mode, most KOs within the pathway shared the same trend. The pathway with consistently increasing (decreasing) KOs would acquire a significantly bigger (smaller) aggregated *Z*-score than the background (Supplementary Figure S1B, blue and red boxes). If KOs within a pathway had opposing trends, the signed *Z*-scores would cancel each other, leading to insignificant results (Supplementary Figure S1B, orange box). In comparison, in the mixed mode, the increasing and decreasing trend of the enriched pathway cannot be determined (Supplementary Figure S1C). Therefore, the directed mode helps find pathways with consistently changing KOs. Several previous studies aimed for the result of the directed mode but mistakenly employed classic RSA (mixed mode) [14].

Another major advantage of GRSA is the full support of multi-group and longitudinal omics data. The ReporterScore package calculates the *P*-value for each feature between groups using differential abundance analysis or correlation analysis. The Kruskal–Wallis test or ANOVA assesses if the feature abundance varies significantly across multiple groups. The default correlation analysis treats group assignments as ordinal (e.g. groups ‘G1’, ‘G2’ and ‘G3’ will be converted to 1, 2 and 3), so the correlation analysis could evaluate if the feature abundance linearly increases or decreases. Moreover, the ReporterScore package also supports any specified patterns. For example, groups ‘G1’, ‘G2’ and ‘G3’ can be set as 1, 10, and 100 if an exponentially increasing trend is expected. To explore potential patterns within the data, clustering methods, such as C-means clustering, can be used.

As a general rule, the users must ensure the selected statistical methods are applicable to the datasets and experimental designs. We applied GRSA with different statistical methods on multiple benchmark datasets. For the classic two-group design, the Jaccard similarity exceeded 0.84 for parametric methods and 0.78 for non-parametric methods, but the Jaccard similarity between parametric and non-parametric methods was lower than 0.63 (Supplementary Figure S2A). The differences mainly stemmed from parametric versus non-parametric methods. For the multi-group data, users can choose the differential abundance analyses if they aim to enrich significantly altered pathways across groups. Correlation analysis is the preferred choice if the goal is to enrich pathways that show consistent increasing or decreasing patterns (Supplementary Figure S2B,C). Lastly, GRSA also supports other statistical tests, such as ‘DESeq2’, ‘Edger’, ‘Limma’, ‘ALDEX’ and ‘ANCOM’ [27], to calculate the reporter scores. The demo code is shown in the Supplementary Notes.

**Figure 2:**
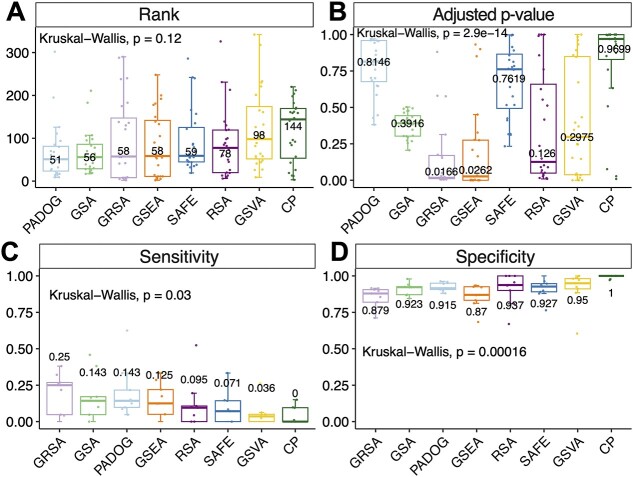
Comparisons of GRSA and other commonly used methods of enrichment analyses. (**A**–**B**) The box charts show the ranks (**A**) and adjusted *P*-values (**B**) of target pathways derived by four methods on 24 gene expression datasets. Numbers represent the median values for each method. (**C**–**D**) The box charts show the sensitivity (**C**) and specificity (**D**) of four methods on nine wild-type/knockout gene expression datasets. Numbers represent the median values for each method. CP: improved Fisher’s exact test used by the clusterProfiler package; GSEA: gene set enrichment analysis; SAFE: significance analysis of function and expression; GSA: gene set analysis in the GSA package; PADOG: pathway analysis with down-weighting of overlapping genes; GSVA: gene set variation analysis; RSA: classic reporter score-based analysis; GRSA: generalized reporter score-based analysis.

### GRSA showed higher sensitivity than other commonly used enrichment analysis methods

Next, we evaluated the performance of GRSA and compared it with other commonly used enrichment analysis methods (Table 1) on several benchmark datasets (Supplementary Tables 2, 3 and the GEO list from ‘GSEABenchmarkeR’ package). PT-based methods may better identify biologically meaningful pathways than non-PT-based methods in certain scenarios [22]. However, PT-based methods require comprehensive topological structures of pathways, limiting their applications in other non-human organisms [28]. Therefore, we focused on the comparison against non-PT enrichment analysis methods. Nguyen et al. proposed several approaches to compare enrichment methods [22], and we adapted their methodologies and evaluated the performance of GRSA against the other popular enrichment analysis methods using the identical pathway database (KEGG v109.0).

We first compared the efficacy of different methods in identifying the target disease pathways using the 24 gene expression datasets associated with known human diseases (Supplementary Table S2). The rationale is that since each dataset is affiliated with a specific disease-linked KEGG pathway (the target pathway), an optimal enrichment analysis method should rank the target pathway among the top of all 342 pathways and enrich the target pathway with a smaller adjusted *P*-value. The results indicate that in terms of assigning a smaller rank to the target pathway, PADOG, GSA, GRSA, GSEA and SAFE performed similarly, as their median ranks all fall within the top 20% (Figure 2A). In addition, GRSA achieves the lowest median adjusted *P*-values of the target pathways (Figure 2B). We also used datasets from a GEO list provided by ‘GSEABenchmarkeR’ for further benchmarking and found similar results (Supplementary Figure S3A, B). Overall, GRSA’s performance is quite favorable among the threshold-free FCS methods, outperforming the conventional ORA method.

We next evaluated the proficiency of different methods in detecting pathways disturbed by gene knockout experiments. In a gene knockout experiment, the knockout gene is a confirmed source of perturbation. Nguyen et al. considered pathways that included the knockout genes as true positives and pathways not including the target genes as true negatives, so the enrichment of such pathways would be considered false positives. Under these assumptions, we could calculate the sensitivity and specificity of a method. GRSA showed the highest median sensitivity among the methods considered, although its specificity is slightly reduced compared to others (Figure 2C, D). We prioritize the sensitivity of a method because for pathways that included the knockout gene, deleting it should have a sizable impact on the pathway (sensitivity); however, for pathways that do not include the knockout gene in the database, given the potential incompleteness of pathway and gene databases, simply attributing these enriched pathways as false positives (specificity) may not always be appropriate.

Lastly, we evaluated the ability of different methods to enrich biologically meaningful pathways [10]. We compared the proportions of pathways identified by the GRSA, the competing tool and both, using the number of all significant pathways as the denominator (Supplementary Figure S3C). GRSA consistently identified a larger proportion of pathways than the ORA method and largely overlapped with GSEA. In these datasets, GRSA identified more enriched pathways than GSEA. For example, in the renal cell carcinoma dataset (GSE6344), pathways related to cytokine−cytokine receptor interaction [29], IL-17 signaling [30] and PI3K–Akt signaling [31] were only enriched by GRSA (Supplementary Figure S4A). In the endometrial cancer dataset (GSE7305), GRSA identified pathways related to cancer, Toll-like receptor [32] and cortisol synthesis [33], which have been shown to be involved in the pathological characteristics of endometrial cancer (Supplementary Figure S4B). Therefore, GRSA can potentially identify more pathways biologically relevant to the studied diseases.

### Case study 1: the functional analysis and age-related dynamics of the skin microbiota

**Figure 3:**
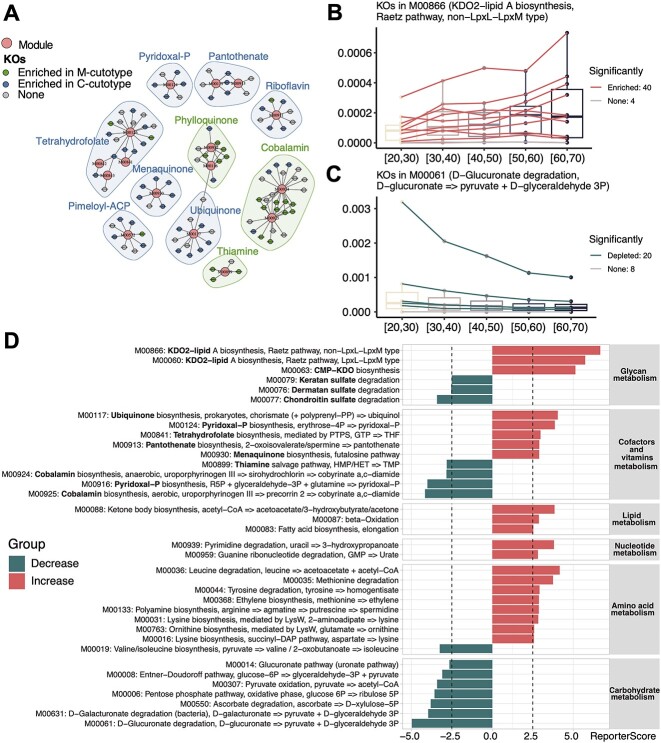
Application of GRSA to the skin microbiome of the IHSMGC dataset. (**A**) The network of KO-Module enriched in the *M-cutotype* (green) and *C-cutotype* (blue). Only modules related to vitamin biosynthesis were shown. Big dots represent modules; small dots represent KOs. The colors of small dots represent cutotypes. Shades indicate modules involved in the biosynthesis of the same vitamin. The colors of shades denote modules enriched in the *M-cutotype* (green) or enriched in the *C-cutotype* (blue). (**B**–**C**) The box charts of modules ‘M00866’ (**B**) and ‘M00061’ (**C**) across ages. The colors of the lines represent the trend of KO’s relative abundance in the module. ‘M00866’ had the biggest positive reporter score (increasing), while ‘M00061’ had the biggest absolute value of negative score (decreasing). (**D**) The bar charts show significantly enriched modules over aging; the reporter score threshold of 2.5 corresponds to a confidence of about 0.995, and these modules are grouped based on the KEGG level B. Colors denote up-regulated (red) or down-regulated (green) modules with aging.

Next, we showcased the versatile applications of GRSA with different types of omics data. For microbiome data, we collected the KO profile of the IHSMGC (integrated Human Skin Microbial Gene Catalog) dataset published by Wang et al. [17] and re-analyzed the data using the GRSA method. The previous study calculated the pathway abundance by aggregating the abundances of features within a pathway and then performed differential abundance analysis. We applied the GRSA to find the functional differences between the two cutotypes. The results were largely consistent. As an example, modules related to the biosynthesis of thiamine, phylloquinone and cobalamin were enriched in the *M-cutotype*, while modules related to tetrahydrofolate, menaquinone, pantothenate and ubiquinone were enriched in the *C-cutotype* (Figure 3A). In addition, the *M-cutotype* was enriched with a large number of modules related to the metabolism of sulfur, phenylacetate (aromatic compound) and amino acids, while the *C-cutotype* was enriched with modules related to carbohydrate metabolism (Supplementary Figure S5). Importantly, GRSA also identified pathways not found in the previous study. The *M-cutotype* was enriched with modules related to nucleotide metabolism, such as the degradation and *de novo* biosynthesis of purine (Supplementary Figure S5), indicating that the *M-cutotype* microbiota may have a higher nucleotide turnover rate and stronger proliferation [34].

The previous study divided samples into five aging groups and found that the prevalence of the *M-cutotype* significantly increased with age. However, they did not perform age-related functional analysis. We re-analyzed the multi-group data using GRSA to explore the functional dynamics related to aging. The larger positive reporter scores indicate that the module had an overall increasing trend with respect to age, such as ‘M00866’, related to lipid A biosynthesis (Figure 3B), while modules with negative reporter scores show an overall decreasing trend with respect to age, such as ‘M00061’, related to d-glucuronate degradation (Figure 3C). We next analyzed the chronological trend of the functional modules at the KEGG level B (Figure 3D), which better reflects the overall metabolic activities of the microbiome. We found that the carbohydrate metabolism activity of the skin microbiota decreased with aging, while the lipid, amino acid and nucleotide metabolism activity increased with aging. These results suggest that the energy sources of the skin microbiota significantly change with aging.

The vitamin biosynthesis-related functional modules also showed differences with respect to aging (Figure 3D). For the glycan metabolism-related functional modules, biosynthesis of KDO2-lipid A and CMP-KDO increased with aging. KDO2-lipid A is an essential lipopolysaccharide (LPS) component in most gram-negative bacteria, which has endotoxin activity and stimulates host immune responses through Toll-like receptor 4 (TLR4) [35]. CMP-KDO is an important intermediate in the synthesis of KDO2-lipid A, and CMP-KDO synthesis is the key rate-limiting step for introducing KDO into LPS [36]. These results suggest that the microbiota of aging skins likely accumulated endotoxins, which can stimulate host inflammation. In addition, we found that degradation pathways of several sulfated glycosaminoglycans (chondroitin sulfate, dermatan sulfate and keratan sulfate) decreased in aging skin. Sulfated glycosaminoglycans play a key role in regulating skin physiology, and there is ample evidence that their properties and functions change over time and with extrinsic skin aging [37, 38]. Total sulfated glycosaminoglycan abundance was reduced in aging skin [39], which may lead to the decreased degrading ability of the skin microbiota for the sulfated glycosaminoglycans.

### Case study 2: the functional transcriptional dynamics during cardiomyocyte differentiation

We applied GRSA to the transcriptomic dataset published by Liu et al. in 2017 [18]. The study used the weighted gene co-expression network analysis (WGCNA) method to analyze the temporal transcriptomic changes during the differentiation of cardiomyocytes from 2 hiPSC lines and 2 hESC lines at 4 timepoints (pluripotent stem cells at day 0, mesoderm at day 2, cardiac mesoderm at day 4 and differentiated cardiomyocytes at day 30). Significant changes were observed in the four stages of differentiation among all cell lines. For example, genes in module 1 were highly expressed only in differentiated cardiomyocytes (stage CM), and their enriched Gene Ontology (GO) terms of Biological Process were related to heart functions, such as regulation of cardiac contraction and muscular system processes. WGCNA did not assume the patterns to be linear so that genes can be only highly expressed at day 2 during mesoderm development, for example.

**Figure 4:**
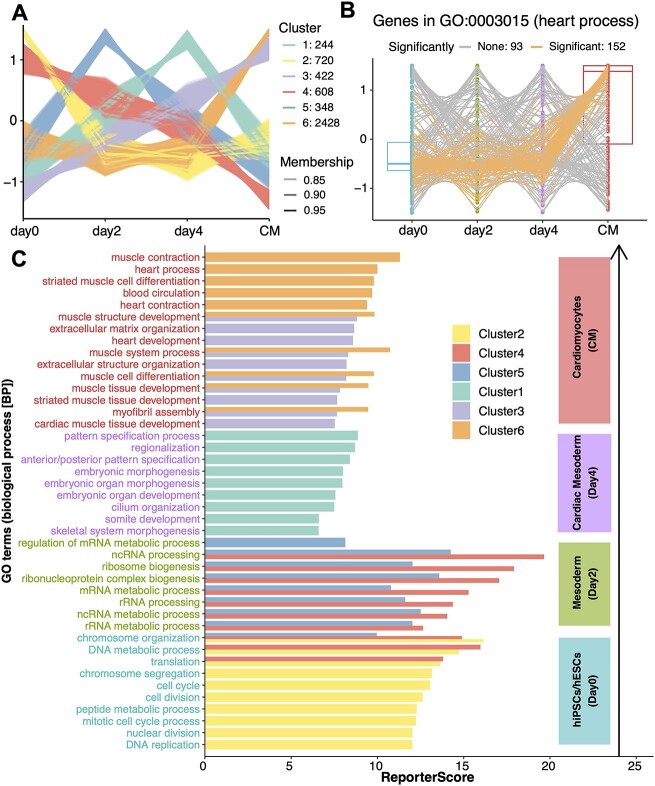
Application of GRSA to the transcriptomic dataset on the cardiomyocyte differentiation processes. (**A**) C-means clustering result of gene abundance profiles across four differentiation stages. The genes with membership scores greater than 0.8 are displayed. The alpha (transparency) of each line was related to the value of its membership score, and the *y*-axis represents the standardized abundance. (**B**) The box chart shows the abundance of genes in ‘GO:0003015’ (heart process) across four time points; the colors of the lines represent the correlative significance of each gene with Cluster 6 within the GO term. ‘GO:0003015’ is a representative term of Cluster 6. (**C**) The bar chart shows significantly enriched GO terms for each clustering pattern corresponding to the differentiation stages. The colors of the bars represent the cluster information, and the representative GO terms with high reporter scores in each cluster are shown. The text labels on the left are colored according to the stages with the highest expression. In general, Cluster 2 corresponds to day 0, Clusters 4 and 5 correspond to day 2, Cluster 1 corresponds to day 4, and Clusters 3 and 6 correspond to CM. Note that only pathways with significant positive scores are shown. The negative score of specified patterns would indicate anti-correlative patterns, which should have already been identified by c-means analysis, such as Cluster 3 versus 4.

In addition to linearly increasing or decreasing patterns, GRSA allows users to specify any expected patterns for enrichment analysis. To start, we used the fuzzy C-means clustering method to identify the main gene expression patterns (Figure 4A) and then used these patterns for GRSA to obtain significantly enriched pathways in each pattern (using the RSA_by_cm function in the ReporterScore package). For example, ‘Heart process (GO:0003015)’ was a significantly enriched GO term for Cluster 6, which was highly expressed only in stage CM (day 30). We identified many genes consistent with the expression pattern of Cluster 6 (Figure 4B).

GRSA results for all clusters are shown in Figure 4C. Cluster 2 was highly expressed only at day 0, and its enriched GO terms were mainly related to the mitotic cell cycle, which was expected for stem cell self-renewal processes. Cluster 5 had the highest expression level on day 2 and was mainly enriched in various transcription and translation processes. Cluster 4 was highly expressed at day 0 and day 2 and showed a gradually decreasing trend; its function overlapped with Clusters 2 and 5. Cluster 1, highly expressed at day 4, was related to mesoderm formation, such as morphogenesis and organ development. Clusters 3 and 6 were primarily up-regulated in differentiated cardiomyocytes (CM stage), and they were related to heart functions, such as regulation of heart contraction and muscle system processes, similar to module 1 in the previous study. Interestingly, the biological processes of hiPSCs/hESCs at day 2 (Cluster 5) induced various RNA-related metabolisms, which were not found in the previous study, indicating that complex transcriptional regulations are involved for further mesoderm formation. Therefore, using the identified expression patterns across groups, we successfully identified pathways and modules essential to different stages of the cardiomyocyte differentiation processes.

### Case study 3: the systematic maternal metabolomic changes correlated with gestational age

We next applied GRSA to metabolomic data from a Danish pregnancy cohort in which female participants had blood drawn weekly from pregnancy to the postpartum period for untargeted metabolomics analysis [16]. Using gestational age as the study variable, they modeled a metabolic clock and found that several marker metabolites increased linearly with gestational age.

**Figure 5:**
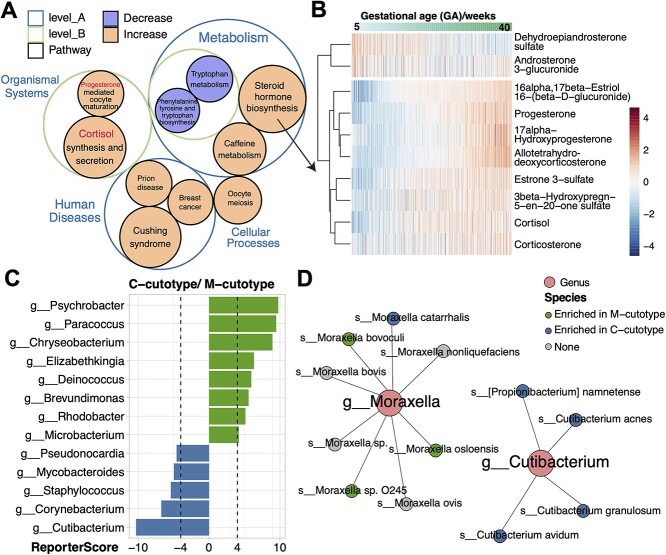
Application of GRSA in the metabolic data of the Danish pregnancy cohort and the taxonomic enrichment analysis of the IHSMGC dataset. (**A**) The circle packing chart shows the hierarchical relationships of significantly enriched pathways identified by GRSA in the metabolomic study. The size of the circle indicates the absolute value of the reporter score, and the color of the circle indicates the sign of the reporter score. The positive reporter score indicates the pathway was increased (orange), while the negative reporter score indicates the pathway was decreased (purple). (**B**) The heatmap shows the abundance of metabolites in the pathway ‘steroid hormone biosynthesis’. The columns are samples ordered by the increasing gestational age. (**C**) The bar chart shows significantly enriched genera in the *C-cutotype* and *M-cutotype*. (**D**) The network plot shows the species in *g_Moraxella* and *g_Cutibacterium* enriched in the *M-cutotype* (green) or *C-cutotype* (blue).

We found several important pathways up-regulated with gestational age: steroid hormone biosynthesis, cortisol synthesis and secretion, and oocyte meiosis (Figure 5A). Multiple steroid hormones were up-regulated with increasing gestational age (Figure 5B), including progesterone that interacts with the hypothalamic–pituitary–adrenal axis [40] and estriol-16-glucuronide produced by the placenta [41]. At the same time, two androgen-related steroid hormones were down-regulated: dehydroepiandrosterone sulfate and androsterone 3-glucuronide, as the concentration of androgens plays important physiological functions during pregnancy [42]. We also found that pathways related to the metabolism of aromatic amino acids were down-regulated with increasing gestational age (Figure 5A), which has been reported [43].

Importantly, we identified several up-regulated pathways related to human diseases not mentioned in the previous study. Cushing syndrome happens when the body has too much of the hormone cortisol for a long time, which could be induced by a healthy pregnancy [44]. The up-regulation of pathways related to breast cancer was also noticeable, as pregnancy-associated breast cancer (PABC) accounts for 7% of all breast cancer in young women [45]. More potential discoveries can be made if the metabolite-pathway database is improved.

### Case study 4: the application of GRSA beyond functional enrichment analysis

The algorithm of GRSA suggests that any features organized in a hierarchical relationship can be used as an enrichment database. For example, we can perform taxonomic enrichment analysis using the phylogenetic relationships of microbes, such as genus–species relationships. To demonstrate, with the custom_modulelist function, we used the species abundance table from the IHSMGC dataset and looked for genera enriched in the two cutotypes. We found that *Psychrobacter*, *Paracoccus*, *Chryseobacterium*, *Elizabethkingia*, *Deinococcus* and *Microbacterium* were enriched in the *M-cutotype*, while *Acidipropionibacterium*, *Staphylococcus*, *Corynebacterium* and *Cutibacterium* were enriched in the *C-cutotype* (Figure 5C), some of which were highly consistent with the differential species modules found by co-occurrence network in the previous study. However, we additionally found some genera, such as *Brevundimonas* and *Rhodobacter*, enriched in the *M-cutotype*, while *Pahexavirus* (phages of *Propionibacterium* and *Cutibacterium*) was enriched in the *C-cutotype* (Figure 5C), probably due to the higher sensitivity of GRSA.

Two species, *Moraxella osloensis* and *Cutibacterium acnes*, were used to define the cutotype in the previous study. Interestingly, while the *Cutibacterium* genus was a good biomarker between cutotypes, the *Moraxella* genus was not, as the included species did not share the same trend (Figure 5D). Therefore, in addition to functional enrichment analysis, GRSA can be extended to any hierarchical relational data structure.

## DISCUSSION

We developed the ReporterScore package and demonstrated broad applications of the GRSA enrichment analyses. We improved the classic RSA method for easier interpretation of the plus and minus signs of the reporter scores. More importantly, we expanded the scope of GRSA from two-group designs to multi-group and longitudinal designs. We demonstrated these new features with metagenomic, transcriptomic and metabolomic data (Figures 3–5). Lastly, we showed that the GRSA is not limited to functional enrichment analysis. Notably, all figures were generated using the visualization module in the ReporterScore package.

GRSA considers all KOs involved in the pathway compared to hypergeometric tests that only consider a pre-defined list (e.g. KO/gene with *P*-value < 0.05). Thus, GRSA is more sensitive and can comprehensively assess the feature abundance differences in the pathway (Figure 2). GRSA still has some limitations: (1) GRSA exhibits a slightly lower specificity, underscoring the necessity for additional experimental validations. (2) GRSA relies on *Z*-scores derived from *P*-values as the statistical measure, which depends on the upstream statistical methods. Users are required to select appropriate statistical methods based on the nature of the data and experimental design. (3) GRSA is a competitive enrichment analysis method that relies on the key assumption of feature independence [46].

We must acknowledge that comparing enrichment analysis methods is fraught with risks, as there is no established gold standard in the field despite continuous efforts [2]. Many reviews typically use disease datasets to evaluate methods based on prioritization or phenotype relevance [8, 23, 28, 47]. We used the ranks and *P*-values of statistical methods to evaluate the prioritization in our comparison (Figure 2A,B), assuming a single true positive: the target disease-associated pathway. However, diseases like cancer cause significant disruptions to complex biological systems. For some diseases, many different pathways could be impacted to varying degrees, with non-target pathways sometimes affected even more than the target pathway [22].

We used the knockout gene experiment datasets to assess sensitivity and specificity referred to Nguyen et al. [22]. We considered sensitivity a crucial metric for enrichment methods because an effective method should maximize the identification of the expected pathways (Figure 2C). The gene–pathway relationships recorded in the database are typically backed by experimental evidence, making the definitions of true positives and false negatives more suitable. Thus, the sensitivity can be effectively evaluated (equation (12)). In contrast, it is difficult to define the true negatives due to the complexity of biological systems leading to the incompleteness of databases. For example, a knocked-out gene may impact pathway A through a signaling pathway yet to be discovered, which frequently happens in molecular biology research. Thus, the specificity of a method cannot be accurately evaluated (equation (13), Figure 2D). Moreover, the primary role of functional enrichment analysis is to guide for interpreting the omics data. Additional experimental verification is essential to substantiate the analysis results.

Multi-omic studies are increasingly prevalent and so are the demands for functional enrichment analysis of all types of omics data [48–51]. The GRSA applies to all types of omics datasets as long as a relevant relational database is available. As demonstrated by case studies, we confirmed previous key findings and acquired new biological insights. For example, applying GRSA on the IHSMGC dataset suggested different functional profiles between aging and young skin microbiota. Biosynthesis of KDO2-lipid A and CMP-KDO increased while degradation pathways of several sulfated glycosaminoglycans decreased in older skin microbiota, which may be linked to changes in the skin’s physiological properties. Further studies are needed to investigate the underlying mechanisms and their implications for skin health. Applying GRSA on the transcriptomic data of cardiomyocyte differentiations revealed that hiPSCs/hESCs at day 2 specialized in various RNA-related metabolisms, suggesting the involvement of complex transcriptional regulation in further mesoderm formation. Finally, applying GRSA to the metabolomic data from the Danish pregnancy cohort showed that several pathways related to human diseases were up-regulated with gestational age, including Cushing syndrome and PABC.

The GRSA offers the option for user-specified patterns for enrichment analysis, allowing for rapid testing of educated hypotheses in complex multi-group studies. This is demonstrated in our analysis of the transcriptomic data during cardiomyocyte differentiations. The GRSA offers applications beyond functional enrichment analysis. Applying GRSA in the taxonomic enrichment analysis of the IHSMGC dataset identified key genera that significantly differed between the two cutotypes. The results were highly consistent with the microbial co-occurrence network analysis in the previous study, but performing GRSA was much easier and faster than the network analysis.

In summary, we believe the GRSA and the ReporterScore package can greatly facilitate the functional enrichment analyses of diverse omics data, with higher sensitivity, compatibility with multi-group and longitudinal designs, and flexibility with customized databases for creative applications beyond functional enrichment analyses. In the future, we plan to incorporate additional built-in databases and visualization techniques, while consistently maintaining and updating the ReporterScore package.

**Key Points**

- We developed generalized reporter score enrichment analysis (GRSA) and the R package ReporterScore, significantly expanding the capabilities of the classic RSA to multi-group and longitudinal experimental designs and is inherently compatible with nearly all types of omics data.
- The GRSA offers higher sensitivity than commonly used enrichment analysis approaches. We confirmed previous key findings and acquired new biological insights in 4 case studies using GRSA.
- The GRSA can be applied to any established relational database and perform customized enrichment analysis, such as taxonomy enrichment analysis.

## Supplementary Material

RS_supp_BIB3-final_bbae116

## Data Availability

Code is available as an open-source R package ‘ReporterScore’ on the Comprehensive R Archive Network (CRAN) (https://cran.r-project.org/web/packages/ReporterScore). The main analysis scripts (Rmarkdown format) and source data are available from GitHub (https://github.com/Asa12138/Analysis_code/tree/main/GRSA_figures).

## Author Biographies

**Chen Peng** is a PhD student in the Life Sciences Institute at Zhejiang University.

**Qiong Chen** is a post-doctoral fellow in the Life Sciences Institute at Zhejiang University.

**Shangjin Tan** is working in the BGI Research.

**Xiaotao Shen** is a post-doctoral fellow in Prof. Michael Snyder’s lab at Stanford University School of Medicine.

**Chao Jiang** is a principal investigator in the Life Sciences Institute at Zhejiang University.
