# Supplementary material for: Generalized reporter score-based enrichment analysis for omics data
Source: Brief Bioinform. 2024 Mar 27;25(3):bbae116. doi: 10.1093/bib/bbae116 (PMC10976918; doi:10.1093/bib/bbae116)
Supplement: RS_supp_BIB3-final_bbae116 [file rs_supp_bib3-final_bbae116.docx]

SUPPLEMENTARY DATA

**Generalized reporter score-based enrichment analysis for omics data**

Chen Peng, Qiong Chen, Shangjin Tan, Xiaotao Shen, Chao Jiang*

# Supplementary Notes

## Demo codes

An example code tailored for one step GRSA of a KO abundance table is as follows.

library(ReporterScore)
# Load the KO abundance table
KO_abundance=read.table("ko_abundance.tsv", header = TRUE, sep = "\t")
# Get the sample metadata
metadata=read.table("sample_metadata.tsv", header = TRUE, sep = "\t")
# Run RSA analysis
reporter_score_res = reporter_score(KO_abundance, "Group", metadata,
 mode="directed", type = "pathway")
# Visualization
plot_report(reporter_score_res, rs_threshold = c(-3,3))
plot_report_circle_packing(reporter_score_res, rs_threshold = c(-3,3))
plot_KOs_in_pathway(reporter_score_res, map_id = "map00780")
plot_KOs_heatmap(reporter_score_res, map_id = "map00780")
plot_KOs_network(reporter_score_res, map_id = c("map05230","map04922"))

An example code for GRSA using other statistical tests is as follows.

#1. Use a specific statistical test method to get the p-values
ko_pvalue=your_method(KO_abundance)
#2. Convert the p-values of KOs to the Z-scores (select mode: mixed, directed)
ko_stat=pvalue2zs(ko_pvalue, mode=choice_of_mode)
#3. Calculate the reporter score of each pathway.
reporter_s=get_reporter_score(ko_stat)

For convenience, the ReporterScore package provides an interface to the above-mentioned enrichment methods: KO_fisher for fisher.test, KO_enrich modified from clusterProfiler based on fisher.test, and KO_gsea modified from GSEA in clusterProfiler. These enrichment methods also support custom databases and are compatible with the format of the input data for the reporter_score function in GRSA, making it easy to make cross-comparisons.

## Benchmark datasets

Benchmark datasets included one metagenomic KO profile (ex_KO_profile downloaded from <https://github.com/wangpeng407/ReporterScore>), 24 gene expression profiles of multiple human tissue types with disease, and 9 gene expression profiles of wild-type/knockout mice from the GEO database (<https://www.ncbi.nlm.nih.gov/geo/>). For further benchmarking analysis, we also included 32 datasets provided by the “GSEABenchmarkeR” package [1].

We used these benchmark datasets with two-group or multi-group experimental designs to investigate the performance of GRSA, including similarities and differences between the two modes, statistical methods, and comparing GRSA with other commonly used enrichment analysis methods. Details of the datasets can be found in Supplementary Table S2 and Supplementary Table S3.

## Case study datasets

Three case studies were re-analyzed using ReporterScore to demonstrate the versatile applications of GRSA, including microbiome, transcriptome, and metabolome.

Skin microbiome data were generated by Wang et al. (2021) [2]. Using the shotgun method, they sequenced 822 skin samples and constructed the complete Human Skin Microbiome Gene Catalog (iHSMGC). A full KO profile based on the KEGG database was provided on the website (<https://ftp.cngb.org/pub/SciRAID/Microbiome>). Metadata with details about including body site, sex, age and cutotype were obtained via <https://static-content.springer.com/esm/art%3A10.1186%2Fs40168-020-00995-7/MediaObjects/40168_2020_995_MOESM2_ESM.xlsx>.

Transcriptomic data were extracted from the study by Liu et al. (2017) [3]. They investigated time-course transcriptomic profiling of cardiomyocyte differentiation derived from human hESCs and hiPSCs. The gene expression matrix is available at <https://www.ncbi.nlm.nih.gov/geo/query/acc.cgi?acc=GSE85331>.

Metabolomic data were generated by Liang et al. (2020) [4]. They analyzed the untargeted mass-spectrum data of 784 samples from 30 pregnant women and built a metabolic clock with five metabolites that time gestational age. The 264 identified level-1 and level-2 metabolites with HMDB IDs and their log2(intensity) can be found at <https://ars.els-cdn.com/content/image/1-s2.0-S009286742030564X-mmc2.xlsx>.

# Supplementary Figures


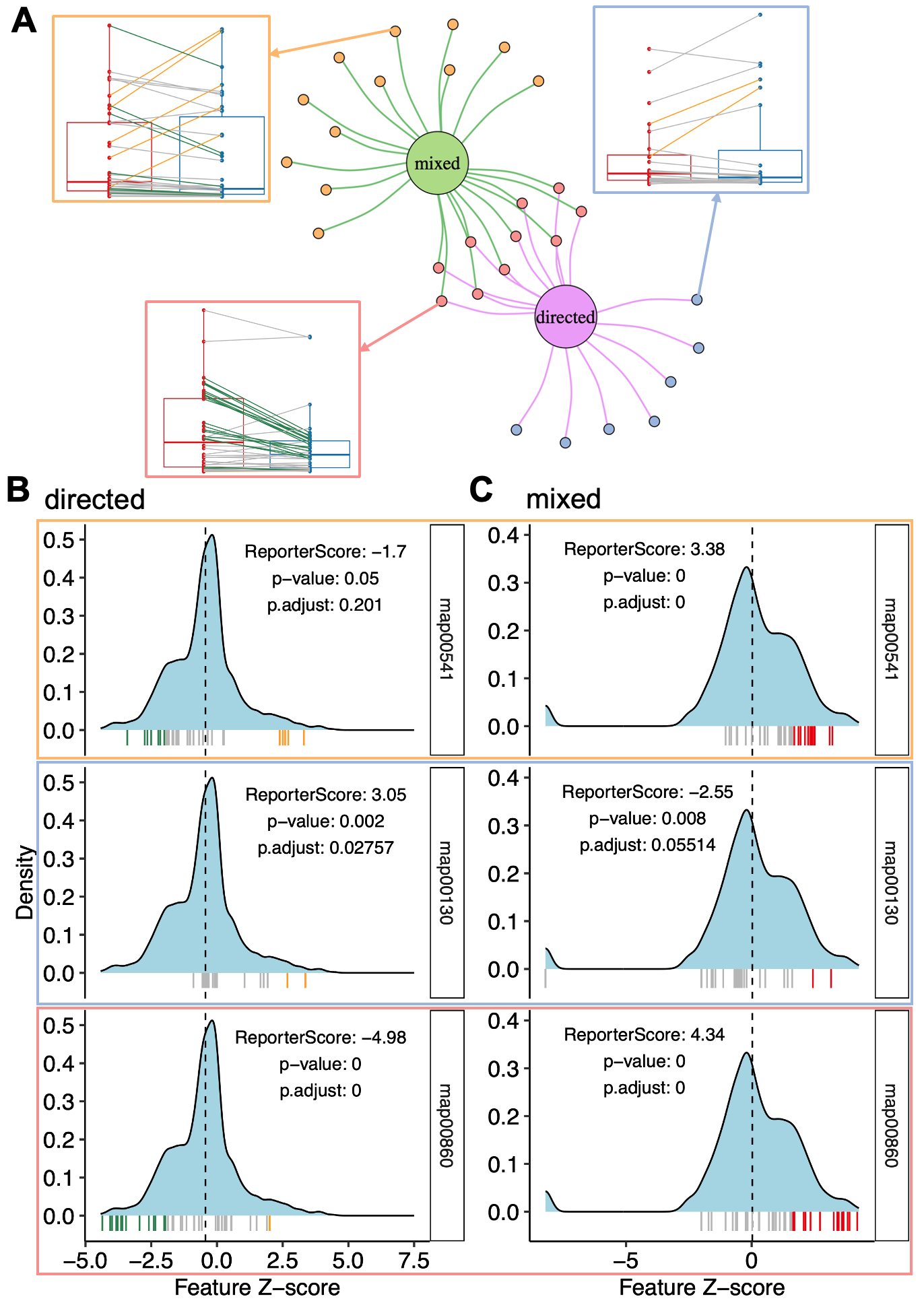


**Supplementary Figure S1.** Differences between the directed and mixed modes of GRSA results of the public ex_KO_profile dataset. (**A**) Venn network of enrichment results using ‘directed’ and ‘mixed’ modes on the ex_KO_profile dataset. Each dot represents a significantly enriched pathway. The box charts show the trends of all KOs within selected pathways. Each line represents the trend of the average abundance of one KO. Line color indicates whether the KO is significantly enriched (orange), depleted (green), or neither (grey). (**B** and **C**) The distribution of KO Z-scores within the selected pathway compared to the background in the directed mode (**B**) and the mixed mode (**C**). The blue shading shows the density distribution of the background Z-score. The bottom whiskers represent the Z-scores of KOs within the selected pathway. The whisker color indicates whether the KO is significantly enriched (orange), depleted (green), or neither (grey).


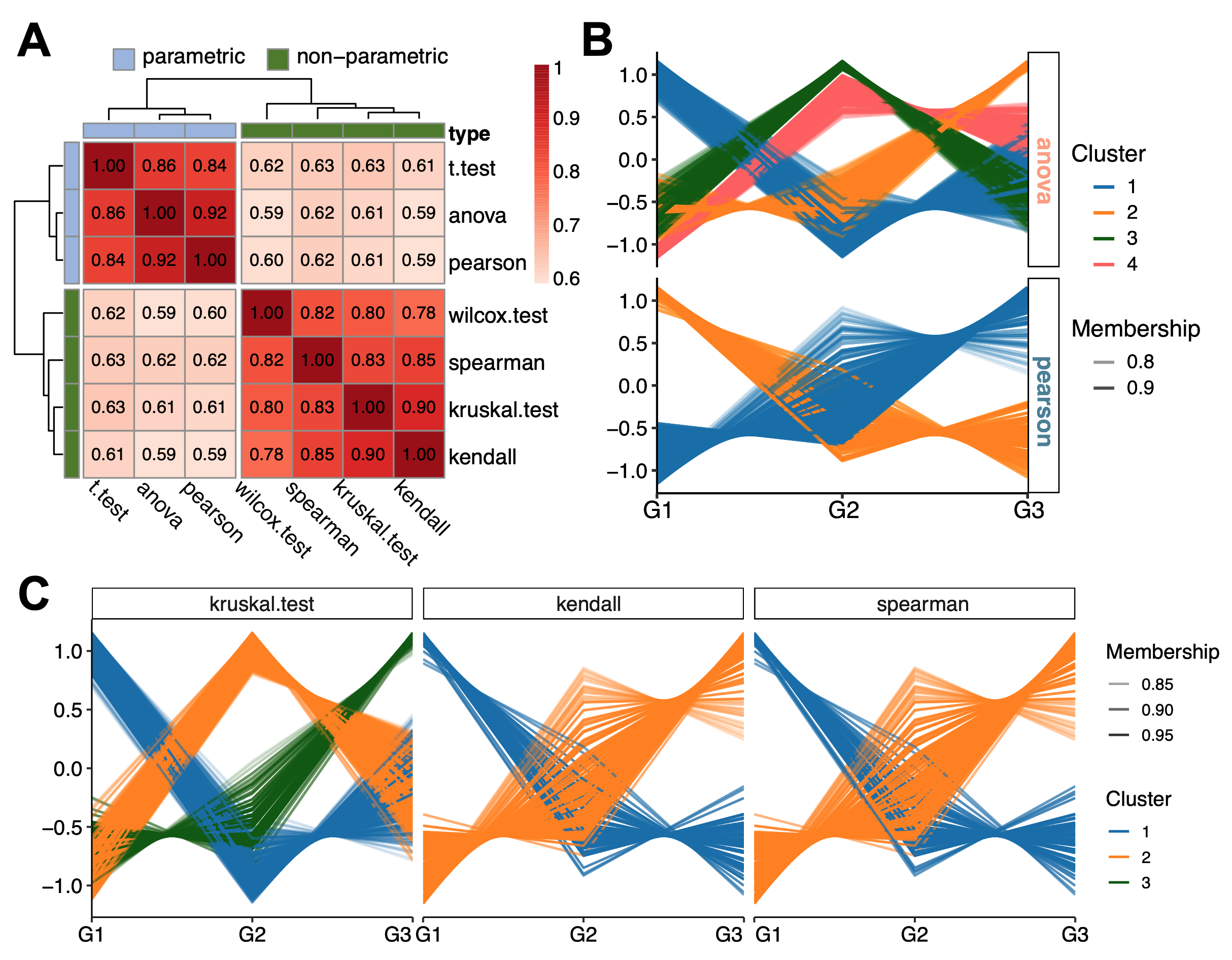


**Supplementary Figure S2.** Impact of the choice of statistical methods on the GRSA results. (**A**) The heatmap shows the average similarity between enrichment results of parametric and non-parametric statistical methods on benchmark datasets. The similarity was defined as the ratio of the number of pathways in the intersection to the union between the two methods. (**B**) C-means clustering of KO features, significance determined by the ‘ANOVA’ and ‘Pearson’ methods (parametric). (**C**) C-means clustering of KO features, significance determined by the ‘Kruskal-Wallis test’, ‘Kendall’, and ‘Spearman’ methods (non-parametric). The alpha (transparency) of each line reflects the value of its membership score, and the abundance was standardized. Only KOs within the significantly enriched pathways were used for the clustering. And only KOs with membership>0.8 were shown.


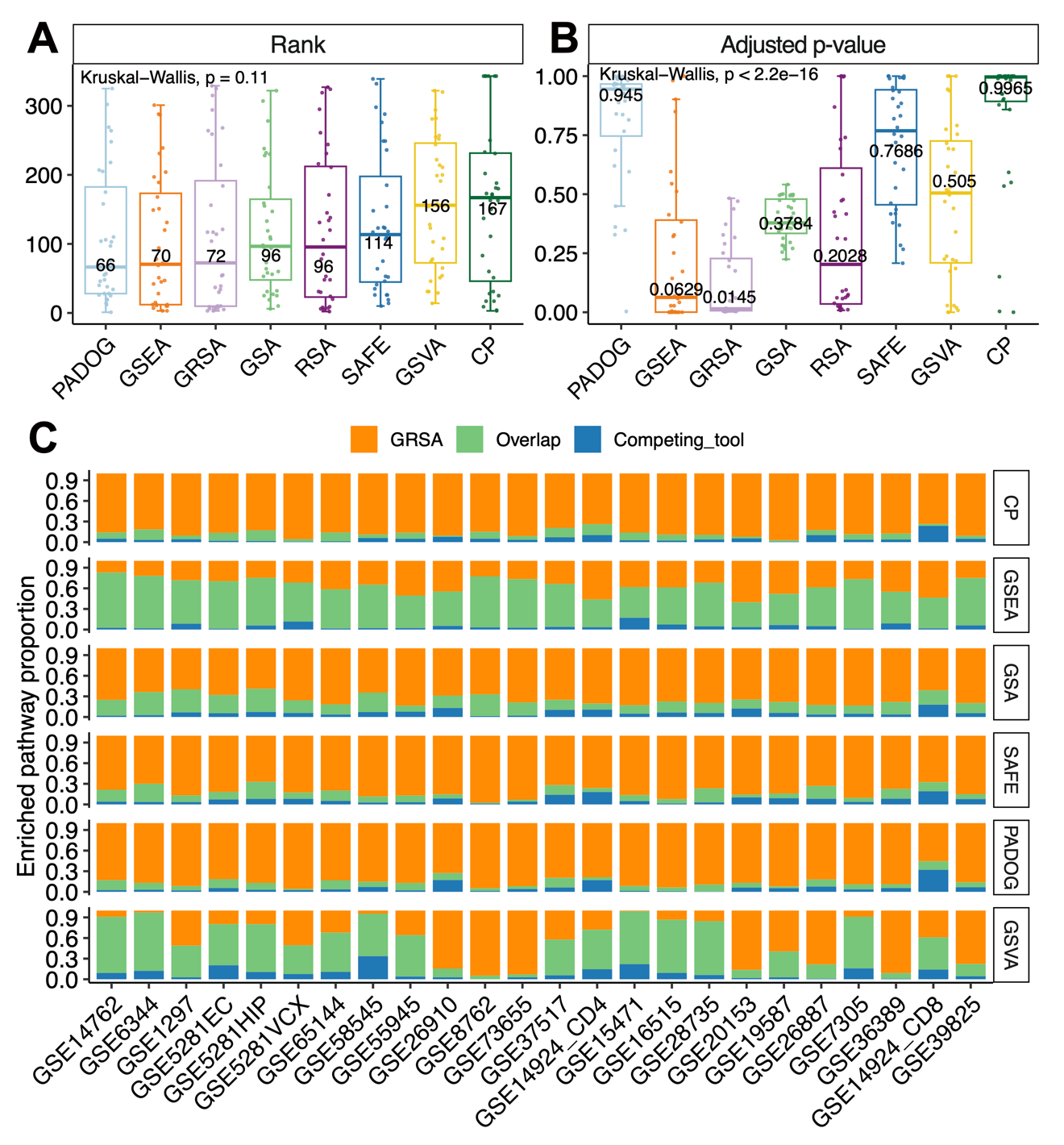


**Supplementary Figure S3.** Comparisons of GRSA and other commonly used methods of enrichment analyses. (**A**-**B**) The box charts show the ranks (**A**) and adjusted *p-values* (**B**) of target pathways derived by four methods on 32 gene expression datasets from the ‘GSEABenchmarkeR’ package. Numbers represent the median values for each method. (**C**) Proportion of enriched pathways in GRSA and other enrichment analysis methods based on 24 gene expression datasets. Orange pathways were only identified by GRSA, blue pathways were only identified by the competing tool and green pathways were identified by GRSA and the competing tool.


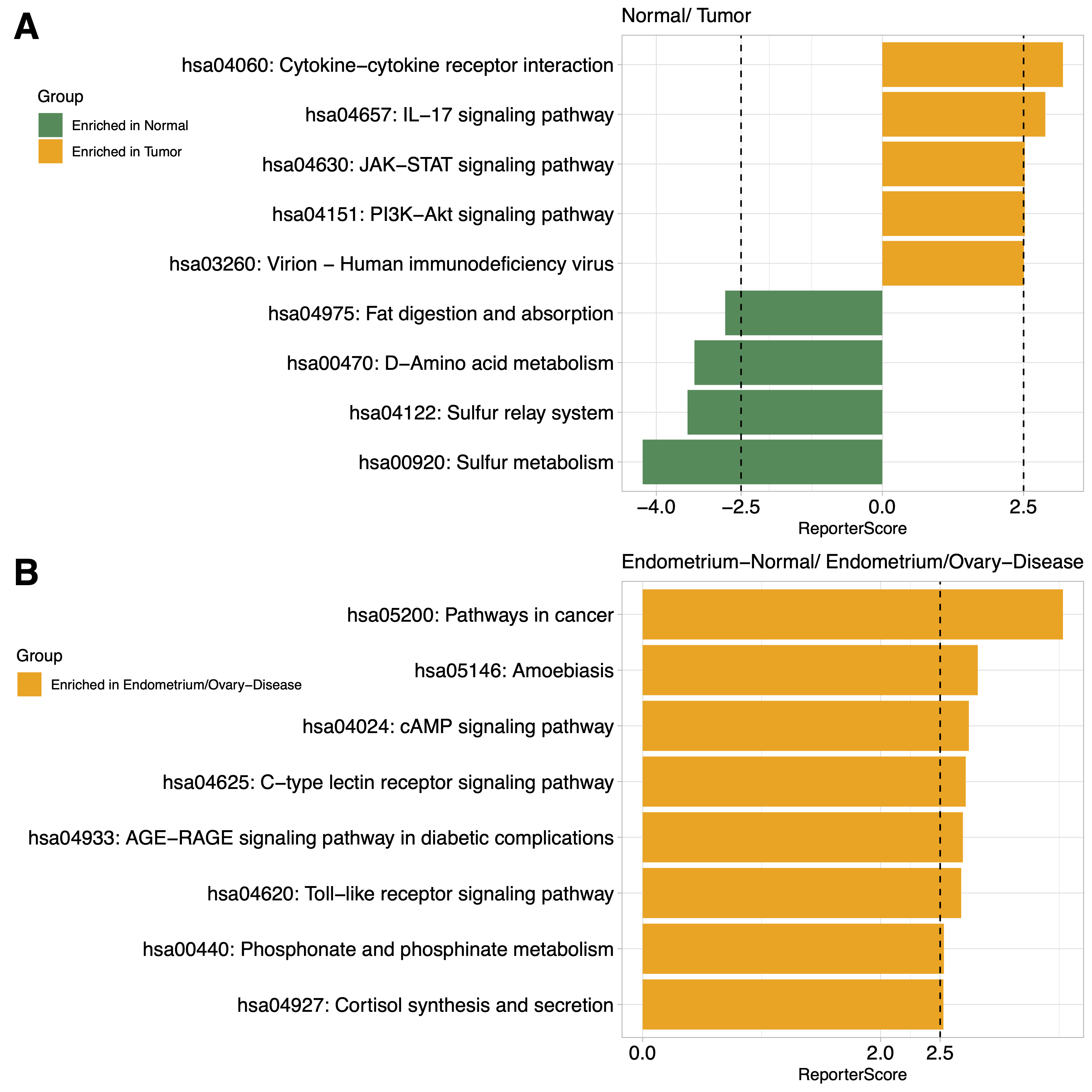


**Supplementary Figure S4.** GRSA-specific enriched pathways compared to GSEA. The Bar chart shows GRSA-specific significantly enriched pathways compared to GSEA in GSE6344 (**A**) and GSE7305 (**B**); the threshold of 2.5 corresponds to a confidence of 0.995. Colors denote the enriched group.


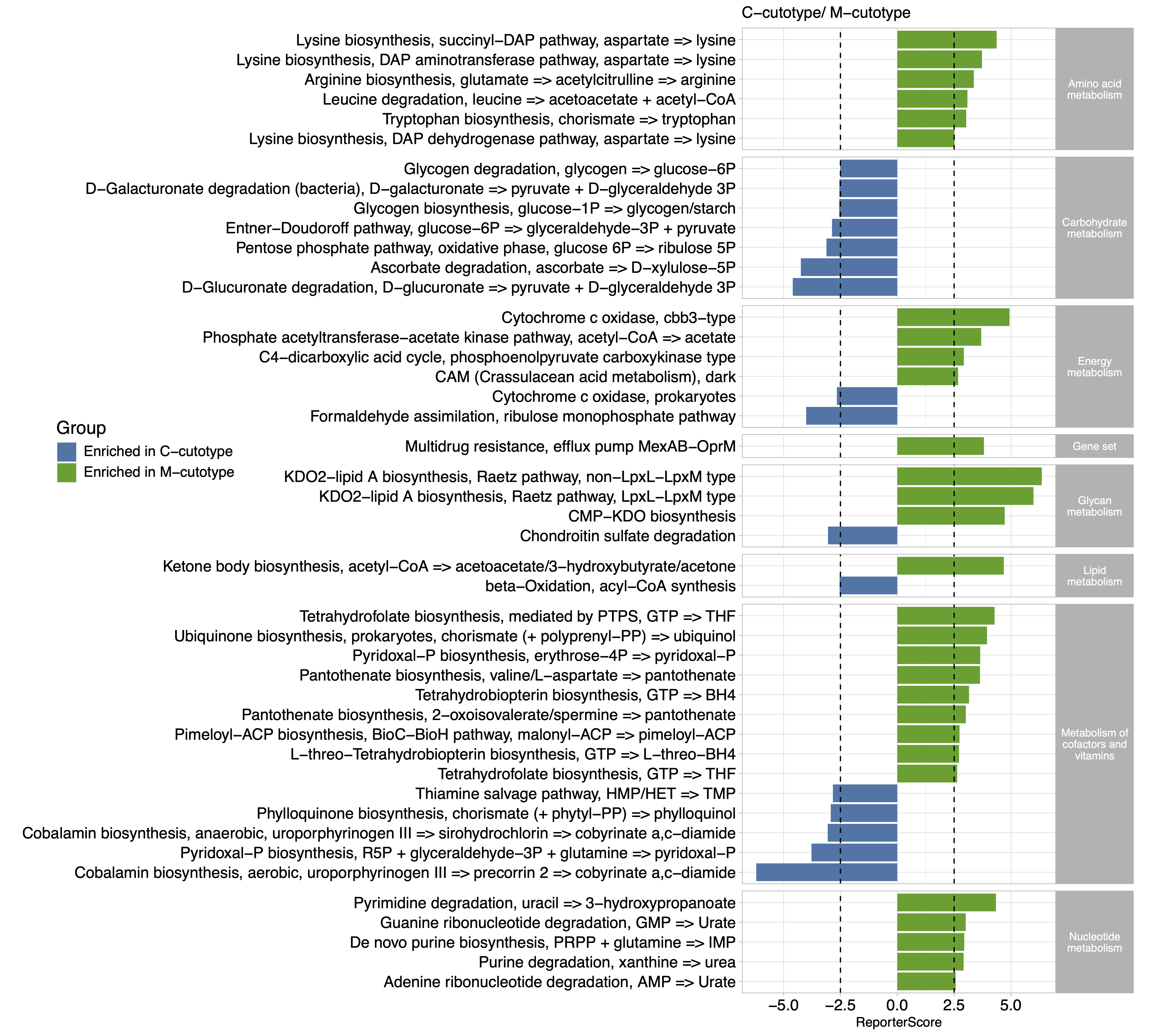


**Supplementary Figure S5.** Significantly enriched modules between *C-cutotype* and *M-cutotype*. The threshold of 2.5 corresponds to a confidence of about 0.995, and these modules are grouped based on the KEGG level B.

# Supplementary Tables

**Supplementary Table S1**. Statistical methods supported by GRSA in the ReporterScore package.

| Method | Analysis | Type | Function |
| --- | --- | --- | --- |
| t-test | differential abundance | parametric | t.test() |
| Wilcoxon rank-sum test | differential abundance | non-parametric | wilcox.test() |
| ANOVA | differential abundance | parametric | anova() |
| Kruskal-Wallis rank-sum test | differential abundance | non-parametric | kruskal.test() |
| Pearson | correlation | parametric | cor.test(method='pearson') |
| Spearman | correlation | non-parametric | cor.test(method='kendall') |
| Kendall | correlation | non-parametric | cor.test(method='spearman') |

**Supplementary Table S2**. 24 benchmark gene expression datasets of 11 diseases used to compare enrichment analysis methods in this study.

| GEO id | Disease | Target pathway | Normal | Case | PubMed | Tissue |
| --- | --- | --- | --- | --- | --- | --- |
| GSE14924_CD4 | Acute Myeloid Leukemia | hsa05221 | 10 | 10 | 19710498 | CD4 T Cell |
| GSE14924_CD8 | Acute Myeloid Leukemia | hsa05221 | 11 | 10 | 19710498 | CD8 T Cell |
| GSE1297 | Alzheimer's disease | hsa05010 | 9 | 7 | 14769913 | Hippocampal CA1 |
| GSE5281EC | Alzheimer's disease | hsa05010 | 13 | 10 | 17077275 | Brain, Entorhinal Cortex |
| GSE5281HIP | Alzheimer's disease | hsa05010 | 13 | 10 | 17077275 | Brain, hippocampus |
| GSE5281VCX | Alzheimer's disease | hsa05010 | 12 | 19 | 17077275 | Brain, primary visual cortex |
| GSE7305 | Endometrial cancer | hsa05213 | 10 | 10 | 17640886 | Endometrium/Ovarian tissue |
| GSE36389 | Endometrial cancer | hsa05213 | 7 | 13 |  | Endometrium |
| GSE8762 | Huntington's disease | hsa05016 | 10 | 12 | 17724341 | Lymphocyte |
| GSE73655 | Huntington's disease | hsa05016 | 7 | 13 | 26756592 | Subcutaneous adipose |
| GSE37517 | Huntington's disease | hsa05016 | 5 | 8 | 22748968 | Neural stem cell |
| GSE15471 | Pancreatic cancer | hsa05212 | 35 | 35 | 19260470 | Pancreas |
| GSE16515 | Pancreatic cancer | hsa05212 | 15 | 15 | 19732725 | Pancreas |
| GSE28735 | Pancreatic cancer | hsa05212 | 45 | 45 | 23918603 | Pancreas |
| GSE20153 | Parkinson's disease | hsa05012 | 8 | 8 | 20926834 | Blymphocytes from peripheral blood |
| GSE19587 | Parkinson's disease | hsa05012 | 10 | 12 | 20837543 | Brain |
| GSE55945 | Prostate cancer | hsa05215 | 7 | 12 | 19737960 | Prostate |
| GSE26910 | Prostate cancer | hsa05215 | 6 | 6 | 21611158 | Prostate |
| GSE14762 | Renal cell carcinoma | hsa05211 | 12 | 9 | 19252501 | Kidney |
| GSE6344 | Renal cell carcinoma | hsa05211 | 10 | 10 | 17699851 | Clear cell RCC |
| GSE65144 | Thyroid cancer | hsa05216 | 13 | 12 | 25675381 | Thyroid |
| GSE58545 | Thyroid cancer | hsa05216 | 18 | 27 | 26625260 | Thyroid |
| GSE26887 | Type II diabetes mellitus | hsa04930 | 5 | 7 | 22427379 | Left ventricle |
| GSE39825 | Type II diabetes mellitus | hsa04930 | 6 | 4 | 23919306 | Fibroblasts (cell culture) |

**Supplementary Table S3**. Nine knockout benchmark gene expression datasets used to compare methods in this study.

| GEO id | Knockout gene | Impacted pathway number | Normal | Case | Pubmed | Tissue |
| --- | --- | --- | --- | --- | --- | --- |
| GSE22873 | Myd88 | 24 | 11 | 8 | 22075646 | Liver |
| GSE70302a | Il1a | 21 | 4 | 4 | 26224856 | Spinal cord |
| GSE70302b | Il1b | 41 | 4 | 4 | 26224856 | Spinal cord |
| GSE58120 | Il2 | 20 | 6 | 6 | 25652593 | Myeloid dendritic cells |
| GSE46211 | Tgfbr2 | 23 | 12 | 6 | 24496627 | Anterior palatal tissue |
| GSE138957 | Akt1 | 97 | 3 | 3 | 32937140 | Cell lines |
| GSE85754 | Fgfr1 | 16 | 4 | 4 | 28433771 | Breast |
| GSE88799 | Crebbp | 28 | 4 | 5 | 28069569 | B cell |
| GSE4451 | Met | 21 | 6 | 6 | 16710476 | Liver |

# References

1. Geistlinger L, Csaba G, Santarelli M, et al. Toward a gold standard for benchmarking gene set enrichment analysis. Briefings in Bioinformatics 2021; 22:545–556

2. Li Z, Xia J, Jiang L, et al. Characterization of the human skin resistome and identification of two microbiota cutotypes. Microbiome 2021; 9:47

3. Liu Q, Jiang C, Xu J, et al. Genome-Wide Temporal Profiling of Transcriptome and Open Chromatin of Early Cardiomyocyte Differentiation Derived From hiPSCs and hESCs. Circulation Research 2017; 121:376–391

4. Liang L, Rasmussen M-LH, Piening B, et al. Metabolic dynamics and prediction of gestational age and time to delivery in pregnant women. Cell 2020; 181:1680-1692.e15
